# Interaction Mechanism of Sulfated Castor Oil in Flotation of Fluorite: Experimental Investigation and DFT Calculations

**DOI:** 10.3390/molecules31214003

**Published:** 2026-11-06

**Authors:** Chunlei Guo, Weiwei Wang, Xuejie Zhang, Burenbayaer Borjigin, Yufang Qin, Jingjing Wang

**Affiliations:** 1 State Key Laboratory of Baiyunobo Rare Earth Resource Researches and Comprehensive Utilization, Baotou Research Institute of Rare Earth, Baotou 014030, China; 2 School of Mining and Coal, Inner Mongolia University of Science and Technology, Baotou 014017, China; 3 School of Resources and Security Engineering, University of Science and Technology Beijing, Beijing 100083, China

**Keywords:** fluorite, sulfated castor oil, flotation, density functional theory

## Abstract

Fluorite (CaF_2_) is a strategically important non-metallic mineral resource worldwide, and flotation remains the predominant technology for its separation from associated gangue minerals. However, conventional fatty acid-based collectors often suffered from limitations such as unsatisfactory separation performance, poor water dispersibility, and unstable flotation performance under complex pulp conditions, necessitating structural modification to improve their collecting capability. Herein, sulfated castor oil (TRO) was proposed as an efficient collector for fluorite flotation, and its flotation performance and interfacial adsorption mechanism were systematically investigated through micro-flotation experiments, zeta potential measurements, X-ray photoelectron spectroscopy (XPS), and density functional theory (DFT) calculations. Micro-flotation tests revealed TRO’s excellent collecting ability for fluorite under weakly acidic conditions. At a TRO concentration of 4 × 10^−4^ mol·L^−1^, the fluorite recovery exceeded 98%, which was significantly higher than that achieved with sodium oleate (NaOl) under comparable single-mineral conditions. Zeta potential measurements indicated that TRO adsorption on fluorite involved both electrostatic interactions and chemical interactions, with the dominant contribution depending on the solution pH. XPS analyses further confirmed the occurrence of chemical adsorption between TRO and fluorite surfaces. DFT calculations revealed that TRO adsorption on the fluorite (111) surface was thermodynamically favorable, with both carboxylate and sulfate ester functional groups participating in coordination with surface calcium sites. Moreover, electronic structure analyses suggested that carboxylate groups exhibited a relatively higher degree of covalent character during coordination, while sulfate ester groups afforded supplementary anchoring effects. This study provides molecular-level insights into the adsorption mechanism of sulfated collectors on fluorite surfaces and offers valuable guidance for the rational design of high-performance collectors for mineral flotation.

## 1. Introduction

Fluorite (CaF_2_) is an important strategic non-metallic mineral resource and serves as an indispensable raw material for the fluorochemical industry. Benefiting from its excellent fluxing properties and unique optical characteristics, fluorite has been widely applied in metallurgy, aerospace, military applications, ceramics, and optical materials [1,2,3,4]. According to mineral composition, fluorite deposits can generally be classified into quartz-fluorite, calcite-fluorite, barite-fluorite, and polymetallic sulfide-associated deposits. However, efficient separation of fluorite from associated gangue minerals remains challenging because fluorite and many gangue minerals exhibit similar physicochemical properties, including comparable density, magnetic response, electrical conductivity, dielectric behavior, and surface characteristics. Therefore, flotation has become the most widely used and effective beneficiation technology for fluorite separation [5,6].

Fatty acids and their derivatives are the most commonly applied collectors in fluorite flotation due to their low cost and strong collecting capability [7,8]. Sodium oleate (NaOl, C_18_H_33_NaO_2_), as a representative fatty acid collector, has been extensively studied and can achieve effective separation of fluorite from quartz and other gangue minerals under suitable flotation conditions. Peng et al. [9] achieved selective separation of fluorite from quartz using NaOl as the collector, with zinc sulfate and sodium silicate as depressants, under both weakly acidic (pH 6.0) and weakly alkaline (pH 9.0) conditions. Nevertheless, the application of conventional fatty acid collectors is still restricted in complex flotation systems because of their intrinsic structural limitations. These collectors generally possess a single carboxyl functional group and long hydrophobic hydrocarbon chains, resulting in drawbacks such as limited aqueous solubility, poor tolerance toward dissolved ions, insufficient selectivity, and decreased flotation efficiency under unfavorable temperature or pulp conditions [2,8]. Previous studies by Miller et al. [10,11] reported that the interfacial interaction between the adjacent oxygen atoms of carboxylate moieties and the fluorite surface can be strengthened with increasing temperature and oxygen concentration, thereby improving fluorite flotation recovery. To overcome these limitations, considerable efforts have been devoted to improving the molecular structure and interfacial activity of fatty acid collectors.

Common modification strategies include halogenation, sulfonation, sulfation, etherification, and nitrogen incorporation, which introduce additional functional groups capable of enhancing molecular polarity, adsorption capacity, and surface activity [4]. Gao et al. [8] found that chlorine atom incorporation not only increases the acidity and reactivity of sodium oleate but also disrupts the hydration film on the fluorite surface due to its large atomic radius, thereby enhancing mineral hydrophobicity. Cao et al. [10] introduced sulfonic acid groups into fatty acid molecules, which effectively improved the surface activity and hard water resistance of the reagents. Moreover, sulfonic acid groups can synergize with carboxylate moieties to coordinate with mineral surfaces and form chelates, further enhancing the adsorption stability of collectors [8]. In addition, the combination of modified fatty acid collectors with other surfactants has been demonstrated as an effective approach to achieve enhanced flotation performance through synergistic adsorption, charge compensation, and regulation of the mineral-solution interface. Schubert et al. [11] adopted oleoyl sarcosine as a collector and successfully obtained acid-grade fluorite concentrate from fluorite ore containing 6% calcite. Their research verified that Ca^2+^ and Fe^2+^ ions, as well as temperatures ranging from 10 to 60 °C, exerted negligible effects on flotation separation efficiency. Apart from single-molecule modification, reagent compounding is another feasible and effective optimization strategy. By rationally blending fatty acid collectors with different types of surfactants at appropriate proportions, synergistic effects can be generated at mineral interfaces through co-adsorption, charge compensation, functional complementarity, and pulp environment regulation, thereby improving overall flotation efficiency. Anionic, cationic, and nonionic surfactants can all be combined with fatty acids to meet the requirements of different flotation systems. Li et al. [12] developed an anionic-cationic mixed collector that delivered pronounced synergistic effects compared with conventional single anionic collectors and effectively improved fluorite flotation. Gao et al. [13] blended NaOl with polyoxyethylene ether to elevate collector surface activity and dispersion, reduce the critical micelle concentration of the flotation system, and further enhance reagent selectivity and operational adaptability. Overall, molecular modification and multi-component compounding have been widely recognized as the primary technical pathways for optimizing the flotation performance of conventional fatty acid collectors.

Sulfated castor oil (TRO, C_18_H_32_Na_2_O_6_S) is a representative anionic surfactant containing both hydrophobic hydrocarbon chains and sulfate ester functional groups. It has been widely utilized in industrial fields including pesticide emulsification, lubrication, fiber treatment, leather processing, cosmetics, detergents, and metal processing [14,15,16]. However, despite its favorable interfacial activity and functional diversity, the potential application of TRO as a mineral flotation collector remains largely unexplored. The coexistence of carboxylate-related active groups and sulfate ester groups in TRO suggests that it may provide additional adsorption sites and stronger interfacial interactions compared with conventional fatty acid collectors, making it a promising candidate for fluorite flotation.

Therefore, we employed TRO as a novel collector for fluorite flotation in this study. Its collecting performance was systematically evaluated through micro-flotation experiments and compared with that of conventional sodium oleate. Furthermore, zeta potential measurements, X-ray photoelectron spectroscopy (XPS) and density functional theory (DFT) calculations were integrated to elucidate the adsorption behavior and molecular interaction mechanism of TRO on fluorite surfaces. The findings provide new insights into the design of high-performance collectors and the molecular regulation of mineral flotation interfaces.

## 2. Results and Discussion

### 2.1. Collecting Performance of TRO

**Figure 1:**
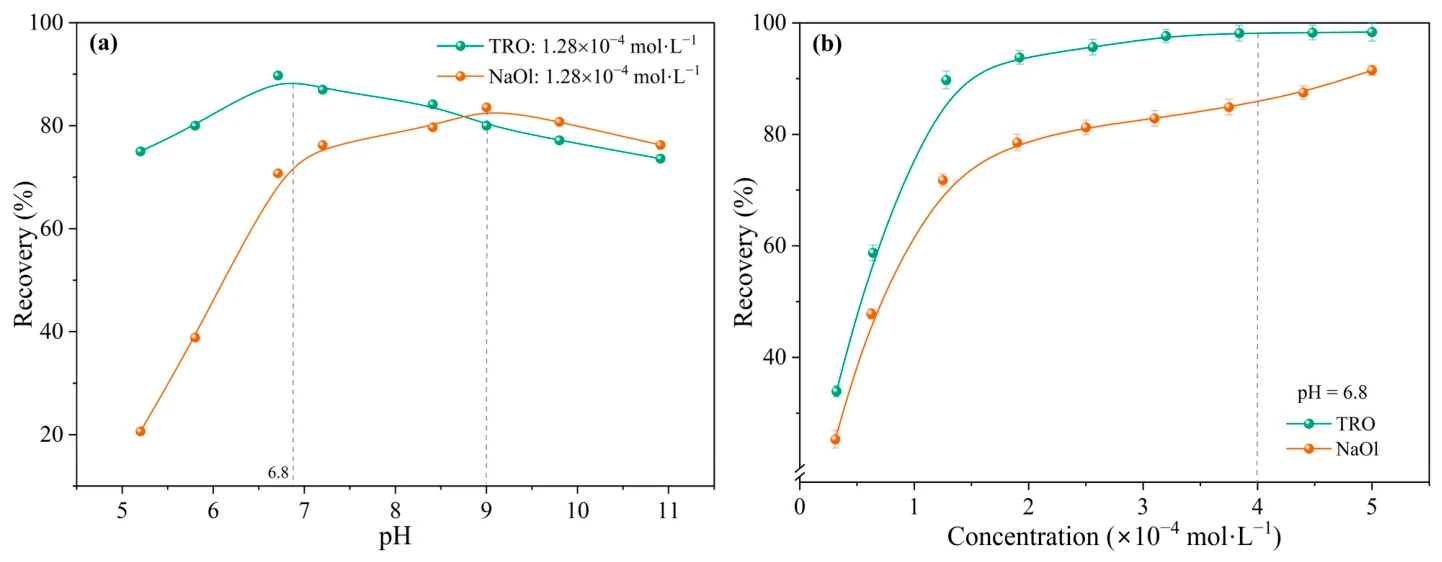
Fluorite flotation recovery as a function of (**a**) pulp pH and (**b**) TRO concentration.

The flotation behavior of mineral collectors is strongly dependent on their chemical speciation in aqueous solution. Therefore, the effect of pulp pH on fluorite flotation performance was first investigated to determine the optimal flotation conditions. The conventional fatty acid collector, NaOl, was selected as a reference reagent for comparison. As shown in Figure 1a, at a fixed collector concentration of 1.28 × 10^−4^ mol·L^−1^, the flotation recoveries of fluorite obtained with both TRO and NaOl exhibited a similar trend, initially increasing and subsequently decreasing with increasing pH. For the TRO system, the maximum fluorite recovery was achieved at pH 6.8, which is comparable to the favorable pH range reported for hematite flotation using NaOl as the collector [17,18]. By contrast, the optimal flotation pH of fluorite with NaOl was 9.0. Notably, TRO exhibited significantly enhanced collecting performance under weakly acidic conditions compared with NaOl, indicating its superior adaptability in low-pH flotation environments. This difference suggests that the introduction of sulfate ester groups into the fatty acid structure may effectively regulate the surface activity and interfacial adsorption behavior of the collector.

Based on the optimal pH condition determined above, the influence of TRO concentration on fluorite flotation recovery was further evaluated (Figure 1b). The fluorite recovery increased continuously with increasing TRO concentration and reached a maximum value exceeding 98% at a TRO concentration of 4 × 10^−4^ mol·L^−1^. In contrast, the maximum recovery obtained with NaOl under comparable conditions was only 84%, demonstrating the superior collecting ability of TRO. Although TRO and NaOl possess similar hydrophobic long-chain structures and carboxylate functional groups, TRO contains additional sulfate ester groups that can provide extra active adsorption sites and strengthen interactions between the collector molecules and the fluorite surface. Therefore, the enhanced flotation performance of TRO may be attributed to the synergistic contribution of multiple functional groups during interfacial adsorption.

It should be noted that the flotation results presented herein were obtained from pure single mineral micro-flotation tests. The data only reflected the collecting capacity of TRO toward fluorite. Separation performance against gangue minerals and practical flotation behaviour in complex ore systems was not evaluated in the current work, which requires further mixed-mineral and real-ore flotation investigations.

### 2.2. Adsorption Mechanism of TRO

#### 2.2.1. Zeta Potential Analysis

**Figure 2:**
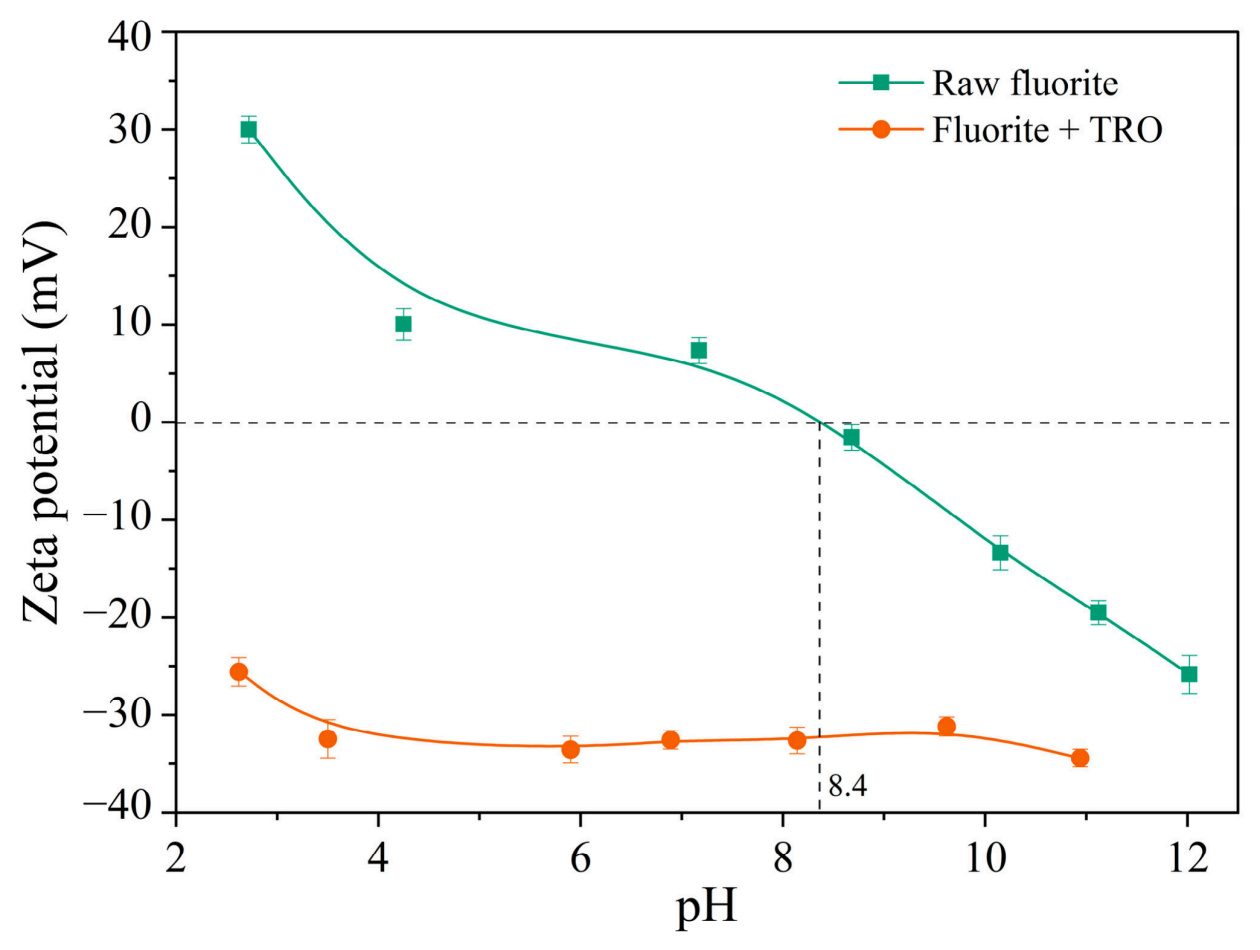
Effect of pulp pH on the zeta potential of fluorite.

Zeta potential measurements were performed to investigate the surface charge evolution of fluorite before and after TRO adsorption and to clarify the interfacial interaction mechanism. As shown in Figure 2, pristine fluorite exhibited an isoelectric point (IEP) of approximately 8.4, which is consistent with previously reported values. The variation in reported fluorite IEP values is mainly associated with differences in mineral origin, impurity composition, and experimental conditions [19,20].

After the addition of TRO, the zeta potentials of fluorite shifted markedly toward more negative values across the entire tested pH range, which confirmed that negatively charged TRO species accumulated on the fluorite surface. After TRO modification, the fluorite surface retained a relatively stable zeta potential of approximately −32 mV, indicative of sustained TRO adsorption. At pH > 8.4 (IEP), both the bare fluorite surface and dissociated TRO species carried negative charge. Accordingly, electrostatic attraction could not account for TRO adsorption under these conditions. The stable adsorption observed despite electrostatic repulsion pointed to specific chemical interactions between the functional groups of TRO and calcium active sites on fluorite. The slight attenuation of the zeta potential shift at high pH was consistent with the decrease in fluorite flotation recovery.

The pH-dependent adsorption trend could be rationalized by the protonation–dissociation equilibrium of the functional groups in TRO. The pK_a_ values of the carboxyl and sulfate ester groups in TRO were predicted to be 4.3 and −4.0, respectively, using MolGpka, a graph convolutional neural network (GCNN)-based web server for pK_a_ estimation. At low pH, carboxyl groups were partially protonated while sulfate ester groups stayed fully ionized. Negatively charged TRO species could then interact with positively charged fluorite surfaces via electrostatic attraction. Near neutral pH, further dissociation of carboxyl groups favored the assembly of molecule–ion aggregates. Such aggregates mitigated electrostatic repulsion and supported robust interfacial adsorption. This behavior coincides with the maximum fluorite flotation recovery at pH 6.8, and analogous molecule-ion dimer formation has been reported for sodium oleate in hematite flotation [18,21,22].

In summary, the zeta potential results revealed the evolution of surface charge on fluorite during TRO adsorption. The adsorption of TRO onto the fluorite surface was driven synergistically by electrostatic interactions and chemical bonding, and the dominant adsorption pathway varied with solution pH. This adsorption mechanism accounted for the excellent flotation performance of TRO under weakly acidic conditions. Nevertheless, these results alone were insufficient to confirm the type of bonding interaction, and additional characterization techniques were required for further verification.

#### 2.2.2. XPS Analysis

**Figure 3:**
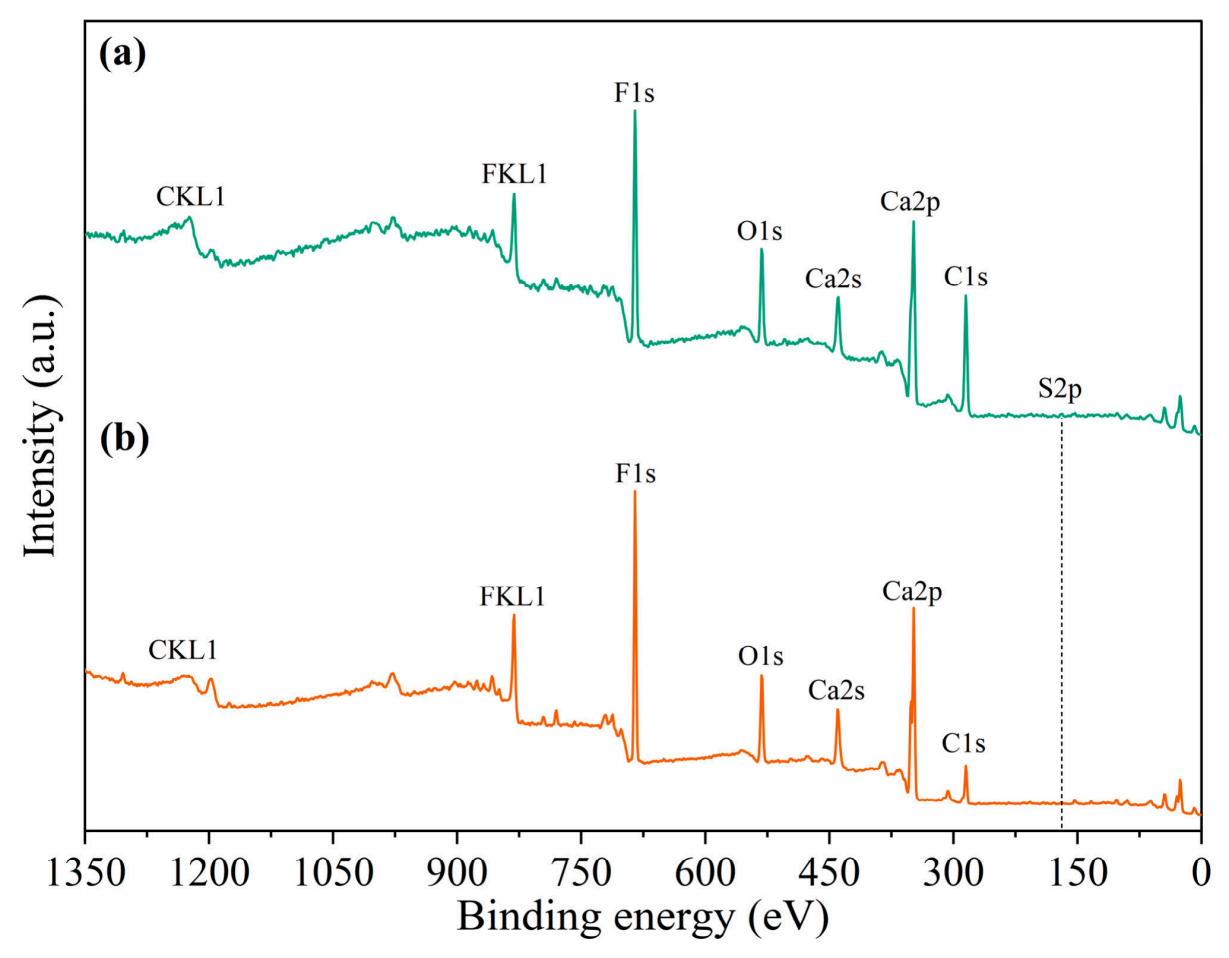
Survey XPS spectra of fluorite before and after TRO treatment: (**a**) fluorite + TRO; (**b**) pristine fluorite.

XPS analysis was further conducted to investigate the surface chemical composition and electronic environment changes in fluorite before and after TRO adsorption. The measurements were performed at the optimum flotation pH of 6.8, where the highest fluorite recovery was obtained. As shown in Figure 3, the pristine fluorite surface mainly consisted of F, Ca, C, and O elements and the normalized relative atomic concentrations of surface elements are summarized in Table 1. After TRO treatment, significant changes in surface elemental composition were observed. The relative atomic concentrations of C 1s and O 1s increased by 31.78% and 8.19%, respectively. Moreover, a new S 2p signal with an atomic concentration of 0.94% appeared on the fluorite surface. The emergence of sulfur species directly confirmed the adsorption of sulfate ester-containing TRO molecules.

**Figure 4:**
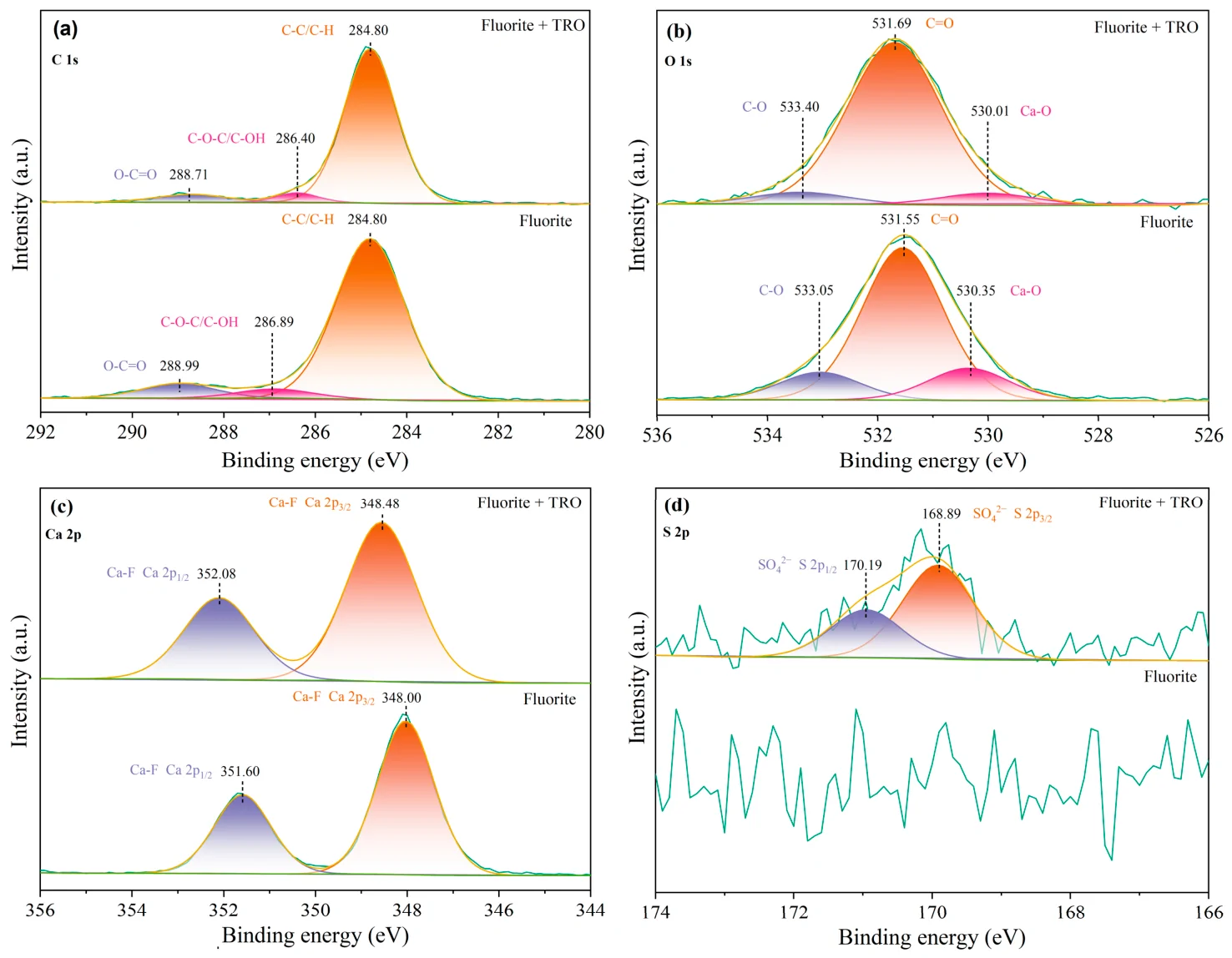
High-resolution XPS spectra of (**a**) C 1s, (**b**) O 1s, (**c**) Ca 2p, and (**d**) S 2p on fluorite surfaces before and after TRO treatment.

High-resolution XPS spectra of C 1s, O 1s, Ca 2p, and S 2p were further analyzed to clarify the chemical interactions between TRO and fluorite. As presented in Figure 4a, the C 1s spectrum of pristine fluorite could be deconvoluted into three characteristic peaks, corresponding to hydrocarbon groups (C-C/C-H, 284.80 eV), hydroxyl/ether groups (C-OH/C-O-C, 286.89 eV), and carbonyl ester groups (O-C=O, 288.99 eV), respectively. These carbon-containing species were mainly attributed to inevitable adventitious carbon contamination introduced during sample preparation and XPS measurement [23,24]. After TRO treatment, the binding energies of C-OH/C-O-C and O-C=O groups shifted positively by 0.49 eV and 0.28 eV, respectively, indicating changes in the local electronic environment caused by interaction between TRO molecules and the fluorite surface. These shifts suggest interfacial electron redistribution during adsorption.

As shown in Figure 4b, the high-resolution O 1s spectrum of pristine fluorite can be deconvoluted into three components, including the Ca-O component at 530.35 eV, the C=O component at 531.55 eV, and the C-O component at 533.05 eV [25,26]. After TRO treatment, noticeable shifts in the binding energies of these O 1s components were observed. Specifically, the Ca-O and C-O peaks shifted positively by 0.34 eV and 0.35 eV, respectively, indicating that oxygen-containing functional groups were involved in surface interaction. Although the assignment of individual oxygen-containing species may involve some uncertainty due to overlapping binding energy regions, the overall spectral evolution provides evidence for chemical interaction between TRO and fluorite [27].

Figure 4c displays the high-resolution Ca 2p XPS spectra of fluorite before and after TRO treatment. For pristine fluorite, the Ca 2p spectrum presented characteristic spin–orbit split doublets with a constant energy separation of 3.60 eV, which were assigned to Ca 2p_3/2_ at 348.00 eV and Ca 2p_1/2_ at 351.60 eV [20,28]. After TRO treatment, both the Ca 2p_3/2_ and Ca 2p_1/2_ peaks positively shifted to 348.48 eV and 352.08 eV, respectively, indicating modification of the electronic environment around surface Ca atoms. This suggests that calcium active sites on fluorite participate in coordination interactions with TRO functional groups.

The high-resolution S 2p spectra of fluorite before and after TRO treatment are presented in Figure 4d. No detectable S 2p signal was observed on the pristine fluorite surface, confirming the absence of sulfur-containing species prior to TRO treatment. After TRO treatment, a characteristic S 2p spin–orbit splitting doublet appeared on the fluorite surface, with peaks located at 168.89 eV (S 2p_3/2_) and 170.19 eV (S 2p_1/2_), separated by an energy interval of 1.30 eV. These characteristic peaks were assigned to the sulfate ester groups from adsorbed TRO species [29]. The emergence of typical S 2p signals provided further evidence for the adsorption of TRO species onto the fluorite surface. Collectively, the XPS results consistently demonstrated that TRO adsorption on fluorite involves chemical interactions between collector functional groups and surface calcium sites. Both carboxylate and sulfate ester groups contribute synergistically to improving the adsorption stability and flotation capability of TRO.

#### 2.2.3. DFT Calculation Analysis

**Figure 5:**
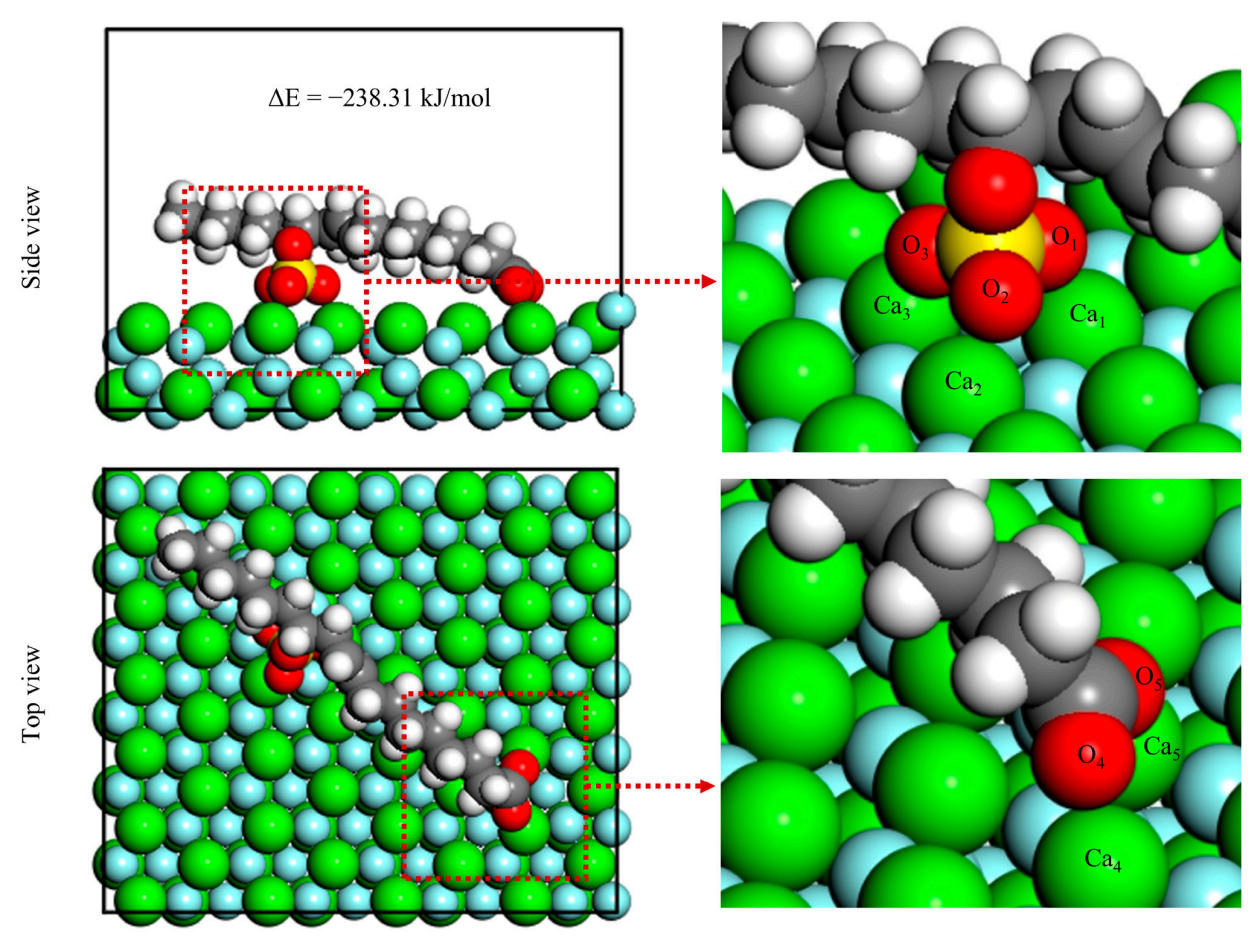
Optimized adsorption configuration of TRO on the fluorite (111) surface (the most common cleavage plane), where green, white, gray-black, red, and light blue represent Ca, H, C, O, and F atoms, respectively.

To further elucidate the molecular-level adsorption mechanism of TRO on fluorite, DFT calculations were performed based on the experimental characterization results. The fluorite (111) surface was selected as the adsorption substrate because it represents the most stable and commonly exposed cleavage plane of fluorite. Under the optimal flotation condition (pH 6.8), TRO species may coexist in molecular and ionic forms in aqueous solution. Considering that ionic species are generally regarded as the dominant active components in flotation systems, the adsorption behavior of ionic TRO on the fluorite (111) surface was investigated. The TRO species was modelled as an anion (TRO^−^). The total charge of the supercell was set to −1, and no Na^+^ counterion was incorporated into the slab model. This approximation assumed that Na^+^ remained in the bulk aqueous solution and did not adsorb on the fluorite surface at pH 6.8. The optimized adsorption configuration of TRO on the fluorite (111) surface is presented in Figure 5. After structural relaxation, the TRO ion adopted a relatively flat adsorption configuration, allowing multiple oxygen-containing functional groups to interact with surface calcium sites. Both carboxylate and sulfate ester groups participated in the interfacial interaction, indicating that TRO adsorption was achieved through synergistic anchoring by multiple functional groups. Specifically, oxygen atoms from TRO coordinated with surface Ca atoms located in the Ca-F six-membered rings of the fluorite surface, forming stable interfacial interactions. The calculated adsorption energy of TRO on the fluorite (111) surface was −238.31 kJ·mol^−1^. The highly negative adsorption energy indicated that the adsorption process is thermodynamically favorable and that strong interactions exist between TRO species and fluorite surfaces. These results confirmed that TRO can form stable adsorption layers on fluorite, which provided a theoretical explanation for its excellent collecting performance observed in flotation experiments.

**Figure 6:**
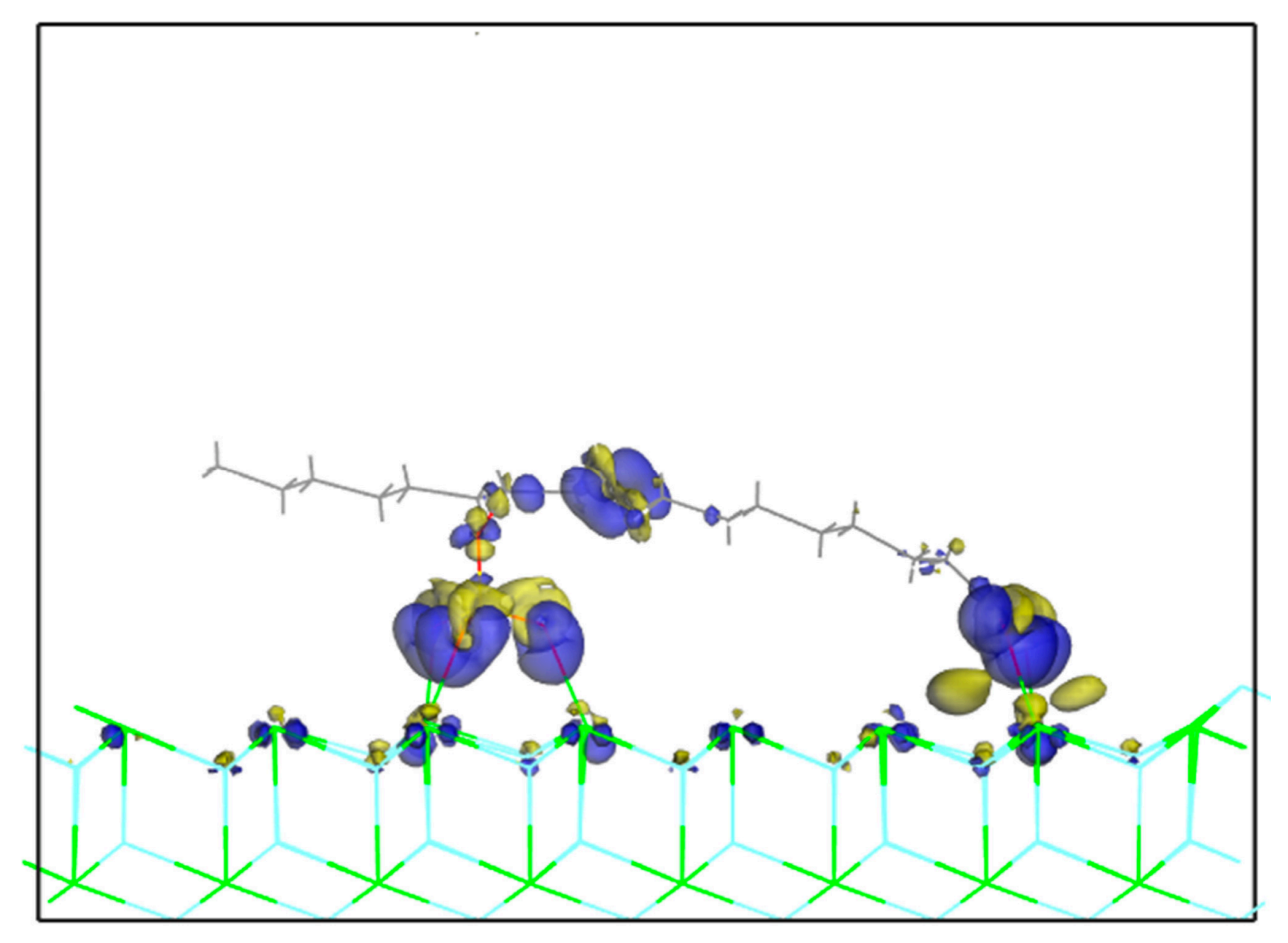
Charge density difference map of TRO adsorbed on the fluorite (111) surface (Isovalue: 0.016), where green, white, gray-black, red, and light blue represent Ca, H, C, O, and F atoms, respectively.

To further understand the electronic interaction during adsorption, the charge density difference in the TRO-fluorite adsorption system was analyzed (Figure 6). The adsorption of TRO on the fluorite surface induced localized charge redistribution and electronic reconstruction at the interfacial region involving O and Ca atoms. Due to the high electronegativity of oxygen atoms (χ_O_ = 3.44, χ_Ca_ = 1.0) [30] in carboxylate groups, electron accumulation regions were mainly concentrated around the O atoms (blue isosurfaces), whereas electron depletion regions appeared around surface Ca atoms (yellow isosurfaces). The paired electron accumulation and depletion features may suggest electron redistribution between TRO oxygen atoms and fluorite calcium sites, lending auxiliary support to the formation of Ca-O coordination interactions. Similar charge redistribution was also observed between sulfate ester oxygen atoms and surface calcium atoms, although the intensity was weaker than that associated with carboxylate groups. This difference indicated that the two functional groups contribute differently to the adsorption process: carboxylate groups act as the primary coordination sites, while sulfate ester groups provide additional interfacial anchoring and improve adsorption stability.

**Figure 7:**
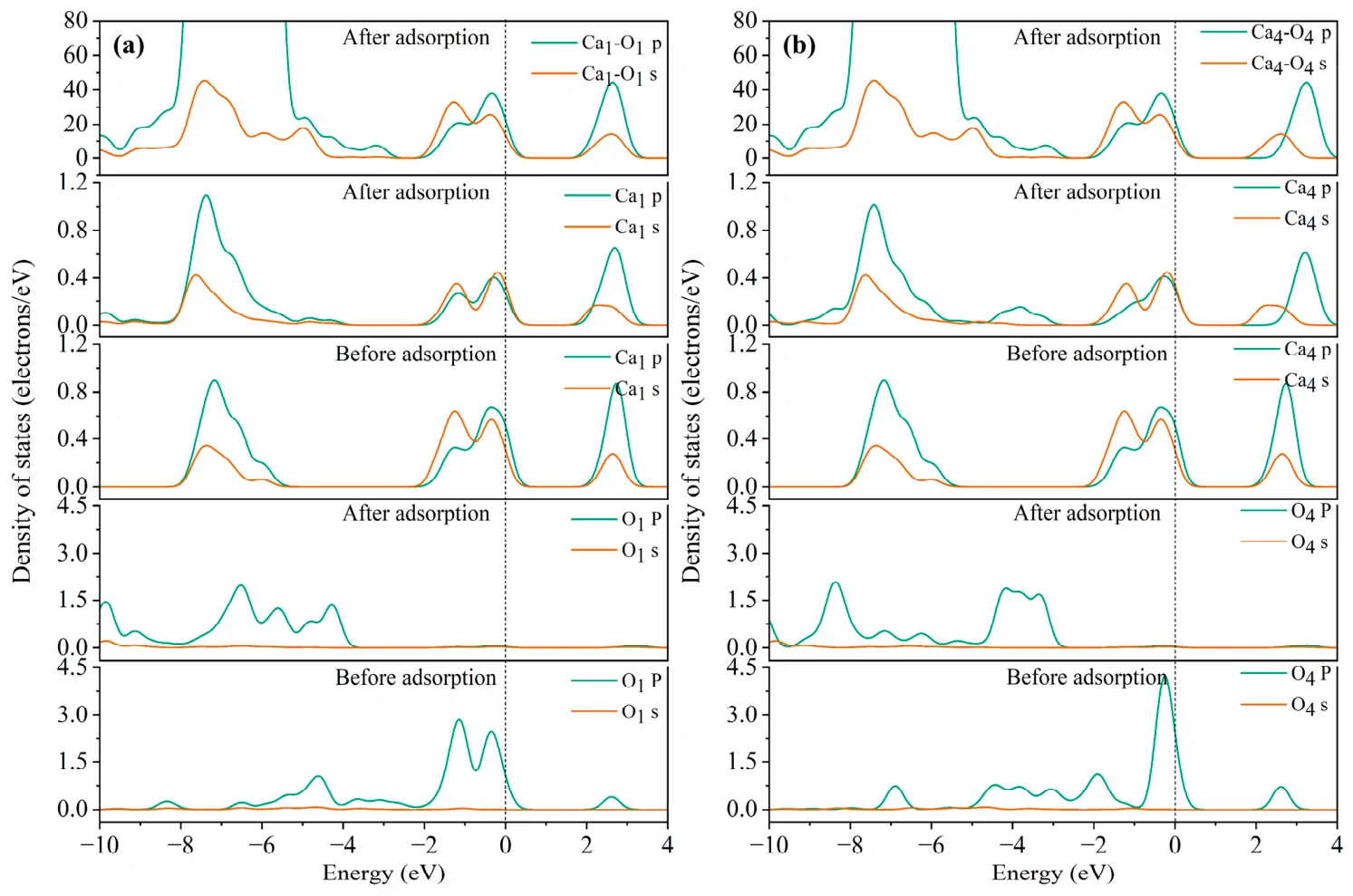
Density of states for the fluorite (111) surface treated by TRO: (**a**) carboxyl oxygen and Ca atoms; (**b**) sulfate ester oxygen and Ca atoms.

The density of states (DOS) analysis was further conducted to investigate the electronic structure evolution before and after TRO treatment (Figure 7). The three oxygen atoms within the sulfate ester group and the two oxygen atoms within the carboxylate group of TRO exhibited resonance-stabilized electronic configurations [31,32]. During the interaction with surface calcium sites of fluorite, O_1_–O_3_ in the sulfate ester group are chemically equivalent, while O_4_ and O_5_ in the carboxylate group also possess equivalent electronic characteristics. Therefore, the DOS distributions of representative O_1_, O_4_, and surface Ca atoms were selected for detailed analysis. Before TRO treatment, the electronic states of surface Ca atoms were mainly distributed around the Fermi level (E = 0 eV) and within the energy range of −8 to −6 eV, with dominant contributions from p orbitals and minor contributions from s orbitals. For the O_1_ atom, the electronic states were primarily located between −6 eV and the Fermi level and were mainly contributed by p orbitals. A broad bimodal feature was observed near the Fermi level, which might reflect a relatively dispersed distribution of electronic states for O_1_ [33]. In comparison, the electronic states of O_4_ were mainly distributed in the range of −5 eV to the Fermi level, also dominated by p orbitals. A sharp single peak appeared near the Fermi level, implying a more concentrated electronic state distribution for the O_4_ atom [34].

After TRO treatment, the intensity of the Ca_4_ p orbital states within the energy range of −8 to −6 eV was significantly enhanced, accompanied by a slight shift toward lower energy levels. Meanwhile, the bimodal feature of the Ca_4_ p states in the −2 to 0 eV region was modified, with slight variations in both peak intensity and position. For the O_1_ atom, the strong bimodal peaks of the p orbital near the Fermi level (−2 to 0 eV) became significantly weakened and broadened after adsorption. Meanwhile, the electronic states in the −6 to −2 eV region were reconstructed, with the appearance of new characteristic peaks, suggesting orbital mixing and interfacial charge redistribution within the adsorption system [35]. For the O_4_ atom, the sharp p orbital peak near the Fermi level (−2 to 0 eV) disappeared after adsorption, accompanied by the emergence of a broadened peak in the −6 to −2 eV region. The electronic structure evolution of O_4_ was more significant than that of O_1_, suggesting that the carboxylate oxygen site (O_4_) exhibited stronger interaction with the fluorite surface than the sulfate ester oxygen site (O_1_). Overall, these systematic changes in the electronic states of surface Ca and O atoms after TRO treatment indicated orbital coupling between Ca and O species, further supporting the coordination interaction at the fluorite-TRO interface [36].

The variations in Hirshfeld charges of oxygen atoms, Ca-O bond lengths, and bond population parameters before and after TRO treatment are summarized in Table 2. As shown in Table 2, all oxygen atoms coordinated with the fluorite surface recorded elevated Hirshfeld charge magnitudes, with increments of 0.099 e, 0.136 e, 0.104 e, 0.144 e, and 0.173 e, respectively. Notably, the O_4_ and O_5_ atoms in the carboxylate group showed slightly larger charge increases than the three oxygen atoms in the sulfate ester group, which implied relatively greater charge redistribution at the carboxylate active sites. Regarding the Ca-O bond lengths, the three oxygen atoms of the sulfate ester group formed bonds with surface Ca atoms at distances of 0.244, 0.242, and 0.251 nm, respectively. The covalent and van der Waals radii of O atoms are 0.066 and 0.152 nm, respectively, whereas those of Ca atoms are 0.176 and 0.231 nm, respectively. Therefore, the O_1_-Ca_1_, O_2_-Ca_2_, and O_3_-Ca_3_ bond lengths fall between the sums of the covalent radii and van der Waals radii of Ca and O atoms, which implies favorable bonding interactions between the sulfate ester groups and the fluorite surface [37]. In contrast, the O_4_-Ca_4_ and O_5_-Ca_5_ bond lengths were calculated to be 0.238 and 0.241 nm, respectively, which are shorter than the sum of the covalent radii of Ca and O atoms. This observation reflected enhanced covalent features for the contacts between carboxylate groups and surface Ca sites. Moreover, the bond population values of O_4_-Ca_4_ and O_5_-Ca_5_ were higher than those of O_1_-Ca_1_, O_2_-Ca_2_, and O_3_-Ca_3_. Within the present computational framework, this trend suggested relatively greater covalent character for the Ca-O contacts involving carboxylate groups. Nevertheless, the bond population values of all interfacial Ca-O bonds were considerably lower than the ideal covalent bond reference value (1.0), indicating that these interactions were not purely covalent but retained significant ionic characteristics. This finding indicated that the adsorption of TRO on the fluorite surface is primarily governed by ionic interactions accompanied by partial covalent contributions, which is consistent with previous studies [38,39].

#### 2.2.4. Adsorption Characteristics of TRO on Fluorite Surface

**Figure 8:**
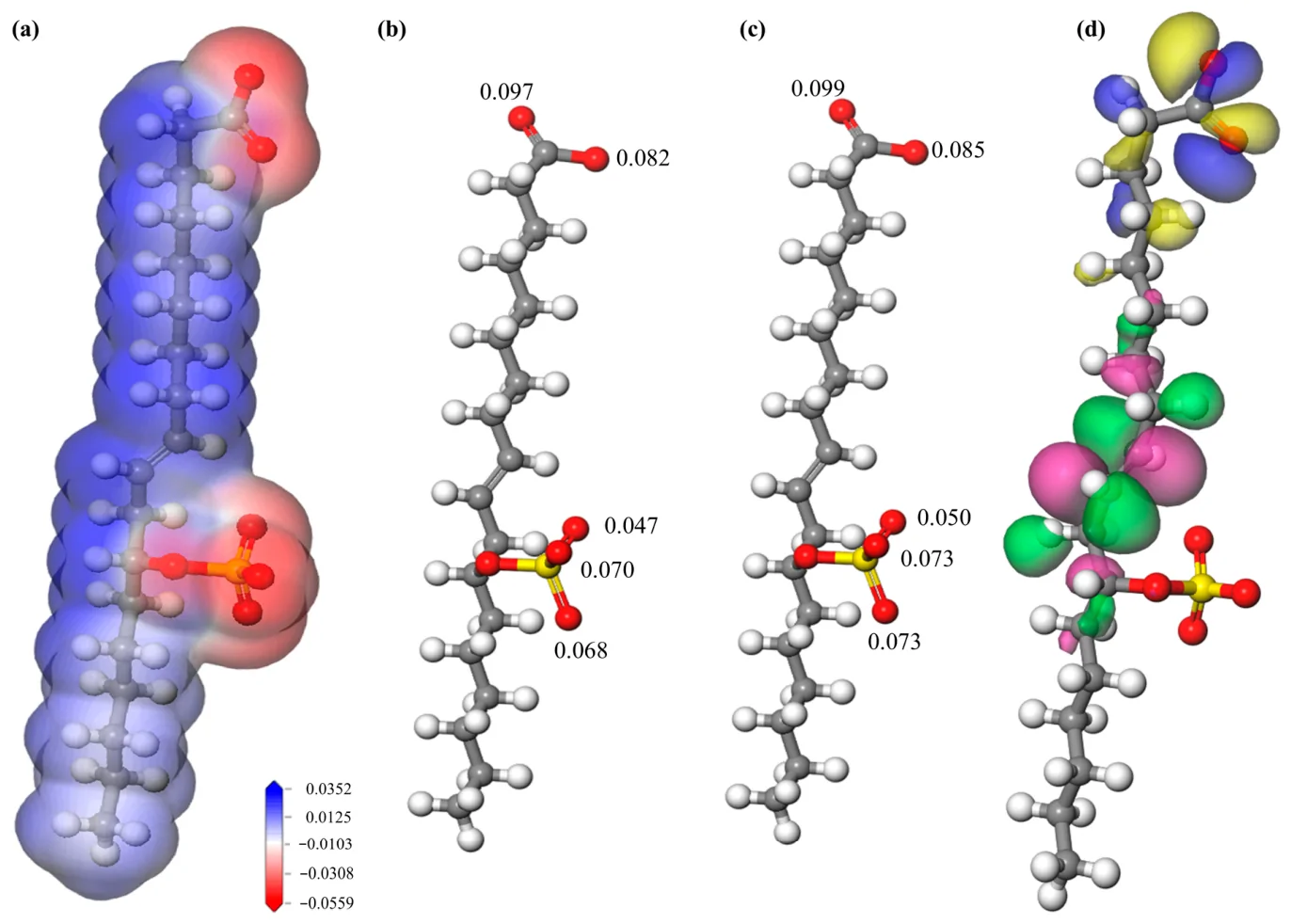
(**a**) Electrostatic potential map, (**b**,**c**) Hirshfeld-based Fukui charge distributions, and (**d**) HOMO and LUMO orbitals of TRO (Isovalue: 0.02; blue and yellow represent HOMO, while green and pink represent LUMO).

Compared with conventional NaOl, TRO contains an additional sulfate ester group besides the common carboxylate functional group, and this structural distinction directly contributes to differences in physicochemical properties and interfacial adsorption behaviors on the fluorite surface. The log *p*-values of TRO and NaOl were calculated as 4.24 and 6.31, respectively, using ACD/ChemSketch software 2025. The lower log *p*-value of TRO suggests enhanced hydrophilicity resulting from the introduction of sulfate ester groups, thereby improving its aqueous solubility. Consequently, TRO can be directly dissolved in flotation solutions without additional emulsification treatment, providing significant operational advantages for practical flotation applications. Furthermore, molecular electronic structure analysis revealed that both the carboxylate and sulfate ester groups exhibited strongly negative electrostatic potential regions (Figure 8a), providing multiple active sites for interaction with the fluorite surface. In contrast, NaOl mainly relies on the carboxylate group for single-site adsorption. The multi-site coordination capability of TRO facilitates the formation of a more stable and densely packed adsorption layer, accompanied by the ordered arrangement of hydrophobic alkyl chains. This enhanced interfacial assembly effectively increases the surface hydrophobicity of fluorite and promotes improved flotation recovery. The superior structural characteristics and corresponding flotation enhancement mechanism of TRO were further confirmed by the micro-flotation results (Figure 1).

In addition, Fukui function analysis (Figure 8b,c) revealed that the oxygen atoms in the carboxylate group of TRO exhibited relatively high Fukui function values, indicating that these atoms possess high local chemical reactivity toward electrophilic and nucleophilic attacks. According to the frontier molecular orbital analysis, the highest occupied molecular orbital (HOMO) of TRO was primarily localized on the carboxylate group, whereas the lowest unoccupied molecular orbital (LUMO) was mainly distributed around the C=C bond of the alkyl chain (Figure 8d). These electronic structure characteristics indicated that the carboxylate group is the most chemically active region of the TRO molecule. Consequently, it can preferentially coordinate with the exposed surface Ca active sites on fluorite, initiating interfacial adsorption and subsequently facilitating the multi-point coordination of the sulfate ester group, thereby enhancing the stability of the overall adsorption configuration.

Meanwhile, compared with conventional single-functional-group collectors, bifunctional collectors bearing two appropriately spaced active groups generally showed better flotation selectivity because of their superior stereochemical and structural compatibility with mineral surfaces [40,41]. The distance between adjacent Ca atoms on the fluorite surface along the same crystallographic direction was calculated to be 0.386 nm, while the O-O distance between the carboxylate and sulfate ester groups in TRO ranged from 1.244 to 1.533 nm, roughly 3–4 times the Ca-Ca spacing on fluorite. This structural matching suggested that the incorporation of sulfate ester groups endowed TRO with greater coordination adaptability to the fluorite surface. Such spatial compatibility served as a favorable structural prerequisite for preferential interaction between TRO and the active Ca sites of fluorite.

## 3. Experimental

### 3.1. Materials and Reagents

**Figure 9:**
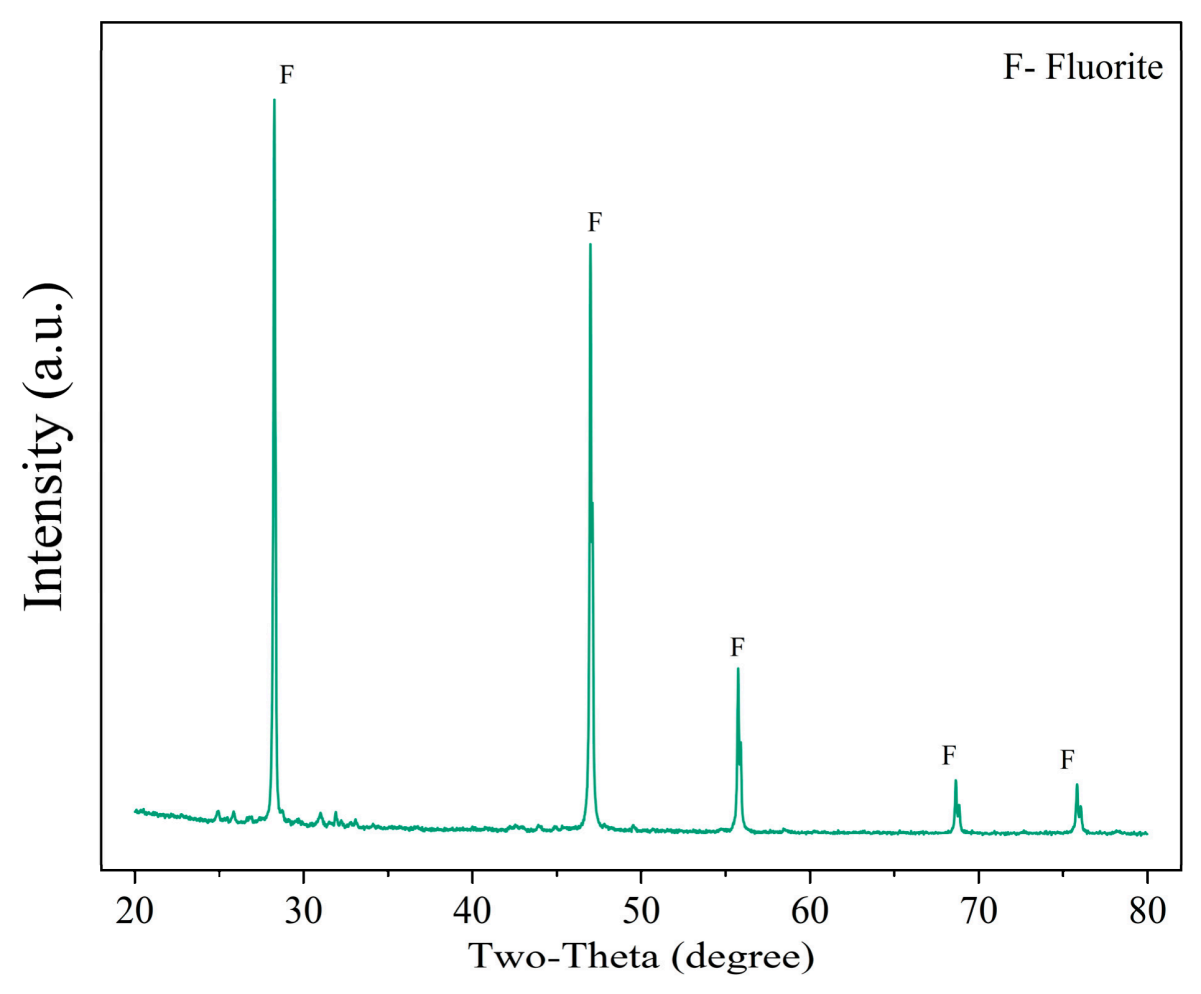
XRD pattern of pure fluorite single mineral sample.

The high-purity fluorite single-mineral sample used in this study was collected from the Bayan Obo mining area, Inner Mongolia, P. R. China. The raw ore was purified through multiple procedures, including crushing, classification, gravity separation, and magnetic separation, to obtain high-purity fluorite specimens. The multi-element chemical composition of the purified fluorite sample is summarized in Table 3, and its phase composition was identified by X-ray diffraction (XRD), as shown in Figure 9.

For micro-flotation experiments, the purified fluorite sample was wet-ground using an XMCQ 180 × 200 porcelain-lined ball mill (Jilin Prospecting Machinery Factory, Changchun, China) and sieved to obtain particles with a size fraction of −38 + 74 μm. For surface characterization analyses, including zeta potential measurements and XPS, the fine fraction (<38 μm) was further ground to below 5 μm using an XQM-100 planetary ball mill (Changsha Tianchuang Powder Technology Co., Ltd., Changsha, China). All grinding tanks and milling balls were made of corundum to minimize contamination.

Both TRO and NaOl were purchased from Shanghai Macklin Biochemical Co., Ltd., Shanghai, China. Hydrochloric acid (HCl) and sodium hydroxide (NaOH) were used as pH regulators, while sodium chloride (NaCl) was employed as the background electrolyte. All analytical-grade reagents used were of purity higher than 98% and supplied by Sinopharm Chemical Reagent Co., Ltd., Shanghai, China. Ultrapure water with a resistivity of 18.25 MΩ·cm was used for all flotation and characterization experiments.

### 3.2. Flotation Experiments

Micro-flotation experiments were conducted using an XFGC aerated hanging-cell flotation machine (Jilin Prospecting Machinery Factory, Changchun, China) equipped with a 40 mL flotation cell. The impeller speed was maintained at 1900 rpm. In each experiment, 2.0 g of purified fluorite sample was dispersed in 40 mL of ultrapure water to prepare a homogeneous suspension. The pulp pH was adjusted to the desired value using dilute HCl or NaOH solution, followed by the addition of collector and conditioning for 3 min. Subsequently, flotation was performed with an air flow rate of 330 mL·min^−1^ for 3 min. Froth products were collected at 10 s intervals. The flotation concentrates and tailings products were separately filtered, dried, and weighed. The fluorite recovery was calculated based on the mass balance of flotation products. All experiments were performed in triplicate, and the average values were reported to ensure experimental reliability and reproducibility.

### 3.3. Zeta Potential Measurements

Zeta potential measurements were carried out using a Nano ZS90 zeta potential analyzer (Malvern Panalytical, Worcestershire, UK) to investigate the surface electrokinetic properties of pristine and TRO-treated fluorite samples. A 1 × 10^−3^ mol·L^−1^ NaCl solution was used as the background electrolyte. A homogeneous mineral suspension with a solid concentration of 0.5 g·L^−1^ was prepared, ultrasonically dispersed for 90 s, and aged for 24 h. Subsequently, 50 mL of the suspension was transferred into a beaker, mixed with a predetermined amount of reagent, and adjusted to the target pH. The suspension was stirred continuously for 30 min and allowed to settle for 10 min before measurement. Approximately 1 mL of supernatant was collected for zeta potential analysis. All measurements were conducted in triplicate.

### 3.4. XPS Measurements

XPS analysis was performed using an ESCALAB QXi X-ray photoelectron spectrometer (Thermo Fisher Scientific, Waltham, MA, USA) to investigate the surface chemical states of fluorite before and after TRO adsorption. The instrument was equipped with a monochromatic Al Kα X-ray source (1486.60 eV) operated at 150 W, and the pressure of the analysis chamber was maintained below 5.5 × 10^−6^ Pa. Before analysis, fluorite suspensions were adjusted to the desired pH, treated with a fixed amount of collector, and magnetically stirred for 2 h. The solid samples were then separated by filtration, washed three times with deionized water under the corresponding pH conditions, and vacuum-dried at room temperature. The obtained XPS spectra were calibrated using the C 1s peak at 284.80 eV to correct for charging effects. Peak fitting and quantitative analysis were performed using Thermo Advantage software v6.9.

### 3.5. DFT Calculation Method

Density functional theory (DFT) calculations were conducted using the Dmol^3^ module in Materials Studio software 2020. The fluorite crystal structure was constructed based on parameters obtained from the American Mineralogist Crystal Structure Database and fully optimized before subsequent calculations. The generalized gradient approximation with the Perdew-Burke-Ernzerhof (GGA-PBE) functional was employed to describe electron exchange-correlation interactions. A 4 × 4 × 4 k-point sampling grid was applied for bulk fluorite calculations. The SCF tolerance was set to 1.0 × 10^−6^ for electron density convergence. The convergence criteria for geometry optimization were 1.0 × 10^−5^ Ha (energy), 2.0 × 10^−3^ Ha/Å (maximum force), and 5.0 × 10^−3^ Å (maximum displacement), respectively. A double numerical plus polarization (DNP 3.5) basis set was adopted with all electrons explicitly included. For surface simulation, a five-layer fluorite (111) slab model with a 15 Å vacuum layer was constructed, and its dimensions were 26.76 Å × 23.18 Å × 19.73 Å. The same optimization parameters were applied, except that the k-point mesh was reduced to 1 × 1 × 1. The TRO molecule was optimized using identical settings. After structural relaxation, electrostatic potential, frontier molecular orbital, density of states, single-point energy, and charge density difference analyses were performed. All calculations were performed under vacuum conditions. Solvent effects, basis set superposition error correction and dispersion correction were not considered in the present simulation. The adsorption energy of TRO on the fluorite surface was calculated according to the following equation:

*ΔE* = *E_TRO-slab_* − *E_TRO_* − *E_slab_*

where *ΔE* represents the adsorption energy, *E_TRO-slab_* is the total energy of the TRO-fluorite adsorption system, and *E_TRO_* and *E_slab_* correspond to the single-point energies of isolated TRO molecules and the pristine fluorite (111) surface, respectively.

## 4. Conclusions

In this work, micro-flotation tests, zeta-potential measurements, XPS and DFT calculations were combined to investigate the flotation performance and interfacial adsorption mechanism of bifunctional sulfated castor oil (TRO) collector for fluorite, with NaOl taken as the reference reagent. Micro-flotation results demonstrated that TRO achieved over 98% fluorite recovery under weakly acidic conditions at pH 6.8, which outperformed NaOl, whose optimal flotation occurred at pH 9.0. Benefiting from the introduced sulfate ester groups, TRO exhibited improved water solubility and required no pre-emulsification.

Zeta potential measurements showed that the surface potential of fluorite shifted significantly toward negative values across the entire tested pH range after TRO adsorption. Even under electrostatic-repulsion conditions at pH > 8.4, TRO still adsorbed stably on fluorite, confirming chemical interactions between the collector and mineral. The molecule-ion aggregates formed under near-neutral conditions weakened electrostatic repulsion, which accounted for the excellent flotation performance of fluorite at pH 6.8. XPS tests verified that both carboxylate and sulfate ester functional groups of TRO underwent chemical coordination with calcium active sites on fluorite at the optimal flotation pH of 6.8. The detection of the S 2p characteristic signal on mineral surfaces further validated the effective adsorption of TRO onto fluorite.

DFT calculations revealed that TRO anions adopted a flat multipoint anchoring configuration on the fluorite (111) surface with an adsorption energy of −238.31 kJ·mol^−1^. The adsorption process was thermodynamically spontaneous and dominated by ionic interactions with partial covalent contributions. Electronic structure analyses indicated that carboxylate groups assumed the primary coordination role, while sulfate ester groups exerted an auxiliary anchoring effect to further enhance adsorption stability. Owing to its bifunctional molecular structure, TRO exhibited favorable structural compatibility with the arrangement of calcium sites on fluorite. Such geometric matching provided a structural basis for preferential interaction between TRO and surface calcium sites of fluorite.

This study uncovers the multi-site synergistic adsorption mechanism of carboxylate-sulfate ester bifunctional collectors and provides theoretical support for the molecular design of high-efficiency fluorite flotation collectors. Further mixed-mineral and real-ore tests are required to evaluate the practical separation performance of TRO.

## Figures and Tables

**Table 1:** Atomic concentration of elements on the fluorite surface as determined by XPS.

Samples	Atomic Concentration (%)				
	C 1s	O 1s	Ca 2p	F 1s	S 2p
Fluorite	4.71	4.17	53.24	37.88	-
Fluorite + TRO	36.49	12.36	28.34	21.85	0.94

**Table 2:** Changes in Hirshfeld charge of O atoms, Ca-O bond lengths, and bond population values.

Atoms	Changs of Hirshfeld Charge on O/e	Bond Lengths/nm	Bond Population
O_1_-Ca_1_	+0.099	0.244	0.25
O_2_-Ca_2_	+0.136	0.242	0.24
O_3_-Ca_3_	+0.104	0.251	0.24
O_4_-Ca_4_	+0.144	0.238	0.30
O_5_-Ca_5_	+0.173	0.241	0.30

**Table 3:** Multi-element chemical analysis of pure fluorite mineral sample (mass fraction, %).

Minerals	CaF_2_	CaO	REO	BaO	TFe	SiO_2_	MgO	Al_2_O_3_
Fluorite	95.34	68.26	1.51	0.98	0.19	0.48	<0.10	<0.10

## Data Availability

The raw experimental data and DFT calculation results that support the findings of this study are available from the corresponding author upon reasonable request.
